# Association of Diabetes and Prognosis of Minor Stroke and Its Subtypes: A Prospective Observational Study

**DOI:** 10.1371/journal.pone.0153178

**Published:** 2016-04-12

**Authors:** Yuesong Pan, Yongjun Wang, Hao Li, Herbert Y. Gaisano, Yilong Wang, Yan He

**Affiliations:** 1 Department of Epidemiology and Health Statistics, School of Public Health, Capital Medical University, Beijing, China; 2 Beijing Municipal Key Laboratory of Clinical Epidemiology, Beijing, China; 3 Department of Neurology, Beijing Tiantan Hospital, Capital Medical University, Beijing, China; 4 China National Clinical Research Center for Neurological Diseases, Beijing, China; 5 Center of Stroke, Beijing Institute for Brain Disorders, Beijing, China; 6 Beijing Key Laboratory of Translational Medicine for Cerebrovascular Disease, Beijing, China; 7 Department of Physiology, University of Toronto, Toronto, Ontario, Canada; Ehime University Graduate School of Medicine, JAPAN

## Abstract

**Background:**

The association between diabetes mellitus (DM) and prognosis of minor stroke is unclear. The aim of this study is to investigate whether DM contributes to the prognosis of minor stroke or its specific subtype.

**Methods:**

All minor ischemic stroke patients were derived from the China National Stroke Registry and classified into 5 subtypes according to the TOAST (Trial of Org 10172 in Acute Stroke Treatment) criteria. DM was defined as either self-reported physician diagnosis of diabetes or use of hypoglycemic medications during hospitalization or at discharge. Patients were followed up for 1 year for clinical outcomes of recurrent stroke, death and functional outcome. Poor functional outcomes were defined as a score of 2–6 for modified Rankin Score. Associations between DM and prognosis of minor stroke and its subtypes were analyzed by univariable and multivariable logistic regression.

**Results:**

Of 4,548 patients with minor stroke, 1,230(27.0%) patients had DM, 1,038(22.8%) had poor outcomes and 570(13.0%) of 4,401 patients had recurrent stroke at 1 year. In multivariable analyses, DM were significantly associated with 1-year stroke recurrence (Odds Ratio [OR], 1.31; 95% confidence interval [CI]: 1.08–1.59) and poor outcome (OR, 1.51; 95%CI: 1.28–1.77). Among the subtypes of minor stroke, DM was only significantly associated with 1-year stroke recurrence (OR, 1.63; 95%CI: 1.07–2.50) and poor outcome (OR, 1.73; 95%CI: 1.22–2.45) in the small-artery occlusion subtype.

**Conclusions:**

DM significantly increased the risk of stroke recurrence and poor outcome in the small-artery occlusion subtype, but not in other subtypes of minor stroke.

## Introduction

Minor ischemic strokes account for approximately 30% of all strokes [1,2]. Although the outcome of an index stroke was favorable with minor symptoms, recent studies have shown a high risk of stroke recurrence and unfavorable outcome within 90 days after the onset of the index event [3,4]. Many predictive scores were established to predict stroke recurrence in patients with general ischemic stroke (such as ESRS score) or transient ischemic attack (TIA) (such as ABCD2 score), but they were not effective in predicting recurrent stroke in minor stroke patients [5]. It is thus necessary to identify the predictors of poor prognosis after a minor stroke.

Most diabetes mellitus (DM) patients are either hyperglycemic and/or exhibit insulin resistance. Both these conditions have been postulated to trigger endothelial dysfunction and atherosclerosis, which contribute to the underlying pathogenesis of stroke [6]. This might have served as rationale for previous studies that showed DM to be an independent risk factor of stroke recurrence after a general ischemic stroke [7–9] or TIA [10,11]. However, studies on minor stroke did not apparently show DM to be an independent risk factor of recurrent ischemic events or poor outcome [2,3]. Whereas, those studies [2,3,7–11] did not distinguish the subtypes of the initial minor stroke. It is now known that ischemic stroke is a heterogeneous disease with variable pathogenesis [12], and DM may be a risk factor for only certain subtypes (not all) of ischemic stroke [13–15]. We here postulate that DM may have differential contribution to prognosis of the different subtypes of minor stroke. Whether DM is an independent risk factor for stroke recurrence or unfavorable outcome after each subtype of minor stroke have not been well studied. Our previous analysis indicated that DM is a predictor for short-term outcomes in patients with a minor stroke [16], the aim of this study is to investigate the association of DM and long-term prognosis of minor stroke and its subtypes.

## Methods

### Study Participants

The study population was derived from the China National Stroke Registry (CNSR) [17]. Details of the rational, design and baseline data of the CNSR have been published previously [17]. In brief, the CNSR is a nationwide, multicenter, prospective hospital-based registry that enrolled patients with a diagnosis of acute cerebrovascular events (≥18 years) within 14 days after onset of stroke. The CNSR study enrolled 22,216 consecutive stroke or transient ischemic attack (TIA) patients from 132 hospitals covering 27 provinces and 4 municipalities across China between September 2007 and August 2008. The protocol and data collection of the CNSR study was approved by ethics committee at Beijing Tiantan Hospital. Written informed consent was given by all patients or his/her representatives before being entered into the study. Patient data in the CNSR data was anonymized and de-identified before the data were accessed for this study.

### Diagnosis of minor stroke and its subtype classification

Acute ischemic stroke was diagnosed according to the World Health Organization criteria [18] with confirmation by brain computerized tomography (CT) or Magnetic Resonance Imaging (MRI). Acute ischemic stroke was diagnosed when the following conditions were met: acute occurrence within 14 days of neurologic deficit, with focal or overall involvement of the nervous system, lasting for >24 hours and after excluding nonvascular causes (primary and metastatic neoplasms, postseizure paralysis, head trauma, etc.) that led to brain function deficit, and excluding intracerebral hemorrhage by computed tomography or magnetic resonance imaging. Acute minor stroke was defined as a score of 3 or less on the National Institutes of Health Stroke Scale (NIHSS) score on admission [2,3].

All ischemic stroke patients were further classified according to the TOAST (Trial of Org 10172 in Acute Stroke Treatment) criteria [19]: large-artery atherosclerosis (LAA), small-artery occlusion (SAO), cardioembolism (CE), other determined pathogenesis and undetermined pathogenesis. Classification of stroke subtype was based on patient’s features combined with the results of 1 or more diagnostic tests, including brain imaging (CT and MRI), electrocardiogram, echocardiography, imaging of extracranial and intracranial arteries, and laboratory assessments for a prothrombotic state. Two neurologists from each participating hospital gave the subtype classifications after detailed review of the clinical features and the results of diagnostic tests. The overall inter-rater agreement for the TOAST classification was good (κ value of 0.73 [95% CI, 0.65–0.81]) [20].

### Data collection and outcome assessment

Baseline data on demographics, status of with or without DM, cardiovascular risk factors and medical treatments, major complications during hospitalization were collected through face-to-face interviews by trained interviewers (neurologists from participating hospitals). DM was defined as a self-reported physician diagnosis of diabetes (including types 1 and 2), use of hypoglycemic medications (eg., insulin, sulfonylureas or biguanides) during hospitalization or at discharge [8]. We also recorded other cardiovascular risk factors, such as hypertension (history of hypertension or anti-hypertensive medication use), dyslipidemia (history of dyslipidemia or lipid-lowering medication use), coronary heart disease, atrial fibrillation (history of atrial fibrillation confirmed by at least one electrocardiogram, or presence of atrial fibrillation during hospitalization), smoking status and heavy alcohol consumption (≥2 standard alcohol beverages per day). We measured stroke severity according to the National Institutes of Health Stroke Scale (NIHSS) score on admission.

We followed up all patients through telephone interview at 3 months, 6 months and 1 year after stroke onset to assess the outcomes of the patients, including recurrence of stroke, death, modified Rankin Score (mRS). The central telephone follow-up was performed by trained interviewers for all patients based on a standardized interview protocol. Recurrent stroke was defined as an aggravated primary neurologic deficit, a new neurological deficit or re-hospitalization with a diagnosis of ischemic stroke, intracerebral hemorrhage, or subarachnoid hemorrhage [18]. All causes of death were collected. Poor functional outcomes were defined as a score of 2–6 for mRS scores [4,21].

### Statistical analysis

For descriptive data analysis, we presented continuous variables as mean±SD or median with interquartile and categorical variables as percentages. We examined the distribution of the baseline variables between patients with DM and those without DM, and also patients with poor prognosis (stroke recurrence, death or poor outcome) and those with good prognosis using t test or Wilcoxon rank sum test for continuous variables and using chi-square test for categorical variables. Baseline variables among different subtypes of stroke were compared by chi-square test for categorical and analysis of variance or Kruskal-Wallis test for continuous variables.

The associations between DM and prognosis of minor stroke or its subtypes were further assessed using univariable and multivariable logistic regression models. All the covariates that showed significant (p<0.05) association with the outcome in the univariable analyses were adjusted in the corresponding multivariable model. Unadjusted and adjusted odds ratios (ORs) and their 95% confidence intervals (CI) were calculated separately. We performed two models, including or excluding random glucose value in the adjustment separately, to show the impact of random glucose value on admission on the results. We then analyzed the association between DM and prognosis of patients in each subtype of stroke. Subgroup analyses of the data by TOAST subtypes were pre-specified.

The α level of significance was p<0.05 two-sided. All analyses were performed with SAS software version 9.3 (SAS Institute Inc, Cary, NC).

## Results

### Characteristics of patient participants

**Fig 1:**
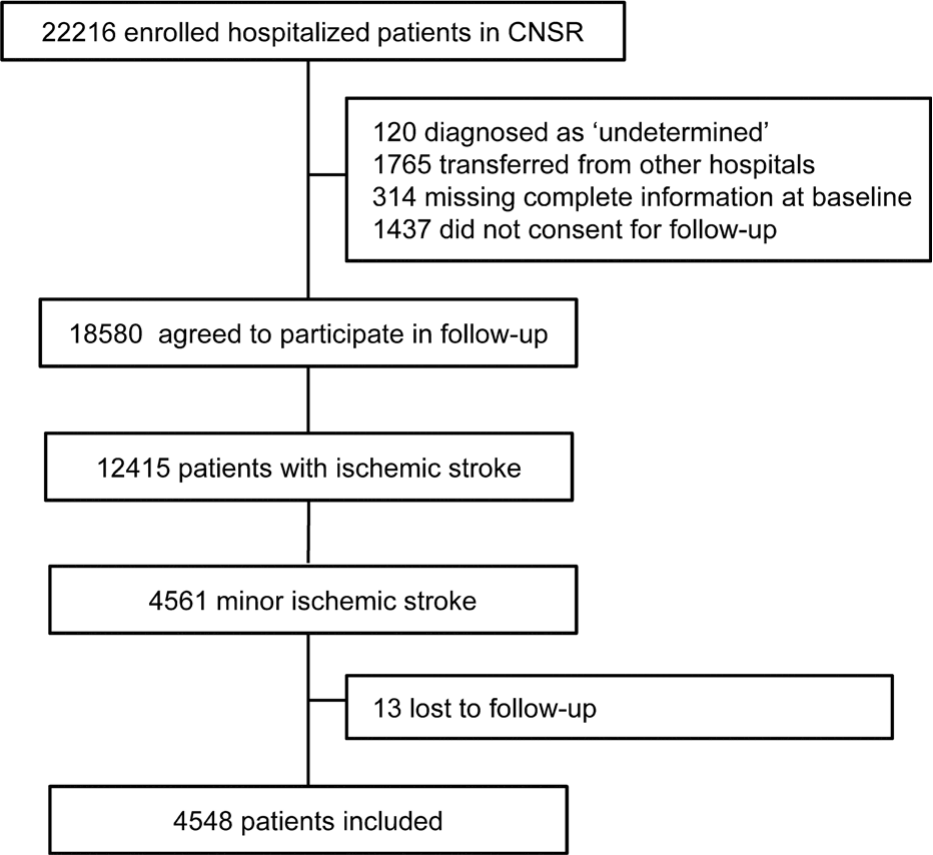
Patient flow diagram.

Of the 22,216 patients enrolled in the CNSR, 4,561 patients with minor ischemic stroke were identified. After excluding 13 patients that were lost to follow-up, 4,548 patients were included in the analysis (Fig 1). Among the 4,548 patients with minor stroke, the average age was 64.1 (range 19–96), and 1,596 (35.1%) were female.

A total of 1,230 (27.0%) minor stroke patients had DM, of whom 961 (78.1%) patients had history of diabetes, and 269 (21.9%) patients were newly diagnosed during hospitalization. The demographics and clinical characteristics of patients with DM and those without DM are presented in Table 1. Compared to those without DM, minor stroke patients with DM were older, more likely to be female, more obese, less smoking and heavy drink, with more severe clinical manifestation (higher NIHSS score) and more vascular risk factors (history of stroke, hypertension, dyslipidemia, coronary heart disease) and complication of urinary tract infection during hospitalization, and were more likely to take antihypertensive agents and statins (Table 1).

**Table 1:** Characteristics of patients with minor stroke with or without diabetes.

Variable	Total (%) (N = 4548)	With diabetes (N = 1230)	Without diabetes (N = 3318)	*p* value
Age (year), mean(SD)	64.1±12.0	65.1±11.0	63.8±12.4	0.003
Female, n(%)	1596(35.1)	488(39.7)	1108(33.4)	<0.001
Body mass index (kg/m^2^), median (IQR)	24.2(22.4–26.3)	25.1(23.1–26.7)	24.3(22.3–26.0)	<0.001
<25	2499(60.0)	583(63.1)	1916(51.9)	<0.001
25–30	1436(34.5)	466(31.9)	970(41.5)	
≥30	227(5.5)	74(5.0)	153(6.6)	
Smoking status, n(%)				<0.001
Never-smoking	2590(57.0)	768(62.4)	1822(54.9)	
Former-smoking	560(12.3)	164(13.3)	396(11.9)	
Current smoking	1289(28.3)	271(22.0)	1018(30.7)	
Unknown	109(2.4)	27(2.2)	82(2.5)	
Heavy drink, n(%)	528(11.6)	111(9.0)	417(12.6)	<0.001
Random glucose value on admission, median (IQR)	6.1(5.3–6.7)	7.1(6.2–10.1)	5.9(5.1–6.4)	<0.001
Pre-stroke mRS >1, n(%)	180(4.0)	66(5.4)	114(3.4)	0.003
NIHSS on admission, median(IQR)	2(1–3)	2(1–3)	2(1–3)	0.008
mean(SD)	1.7±1.0	1.8±1.1	1.7±1.0	
Subtype of stroke*, n(%)				<0.001
Large-artery atherosclerosis	1915(42.1)	567(46.1)	1348(40.6)	
Small-artery occlusion	1071(23.6)	304(24.7)	767(23.1)	
Cardioembolism	115(2.5)	23(1.9)	92(2.8)	
Other or undetermined	163(3.6)	38(3.1)	125(3.8)	
Unknown	1284(28.2)	298(24.2)	986(29.7)	
History of disease, n(%)				
Diabetes	961(21.1)	961(78.1)	0(0.0)	-
Stroke	1365(30.0)	419(34.1)	946(28.5)	<0.001
Hypertension	2905(63.9)	893(72.6)	2012(60.6)	<0.001
Dyslipidemia	510(11.2)	205(16.7)	305(9.2)	<0.001
Coronary heart disease	566(12.5)	196(15.9)	370(11.2)	<0.001
Atrial fibrillation	239(5.3)	56(4.6)	183(5.5)	0.20
Medications during hospitalization, n(%)				
Hypoglycemic^†^	981(21.6)	981(79.8)	0(0.0)	-
Antihypertensive	2095(46.1)	652(53.0)	1443(43.5)	<0.001
Statins	2089(45.9)	642(52.2)	1447(43.6)	<0.001
Antiplatelet	3412(75.0)	941(76.5)	2471(74.5)	0.16
Anticoagulation	56(1.2)	12(1.0)	44(1.3)	0.34
Swallowing assessment, n(%)	1624(35.7)	450(36.6)	1174(35.4)	0.45
Pneumonia, n(%)	215(4.7)	70(5.7)	145(4.4)	0.06
Urinary tract infection, n(%)	70(1.5)	29(2.4)	41(1.2)	0.006

IQR, interquartile range; mRS, modified Rankin Scale; NIHSS, National Institutes of Health stroke scale; and SD, standard deviation.

* Stroke subtype was defined by the Trial of Org 10172 in Acute Stroke Treatment (TOAST) classification.

^†^ Hypoglycemic medications includes use of insulin or oral hypoglycemic agents.

### DM associated with poor prognosis of minor stroke

Among the 4,548 patients with minor stroke, 225 (4.9%) were deceased at 1-year follow-up and 1,038 (22.8%) had poor functional outcomes (mRS 2–6). 4,401 (96.8%) patients had data of stroke recurrence, of whom 570 (13.0%) patients had recurrent stroke at 1-year after index stroke onset.

Univariable analyses of risk factors for poor prognosis of minor stroke are presented in Table 2. Table 3 shows that minor stroke patients with DM had a significantly higher frequency of stroke recurrence and poor outcome both at 3 months and 1 year after index stroke onset. After adjustment for potential confounders, DM was significantly associated with poor outcome at 3 months (OR, 1.46; 95%CI: 1.24–1.71), stroke recurrence (OR, 1.31; 95%CI: 1.08–1.59) and poor outcome (OR, 1.51; 95%CI: 1.28–1.77) at 1 year (Table 3).

**Table 2:** Characteristics of patients with minor stroke according to the 1-year outcomes.

Variable	Stroke	recurrence			Death		Poor	Outcome	
	Yes (N = 570)	No (N = 3831)	p value	Yes (N = 225)	No (N = 4323)	p value	Yes (N = 1038)	No (N = 3510)	p value
Age (year), mean(SD)	67.0±11.9	63.7±12.0	<0.001	71.3±12.0	63.7±11.9	<0.001	70.1±10.8	62.3±11.8	<0.001
Female, n(%)	198(34.7)	1350(35.2)	0.81	78(34.7)	1518(35.1)	0.89	422(40.7)	1174(33.5)	<0.001
Body mass index (kg/m^2^), median (IQR)	24.5(22.5–26.6)	24.2(22.3–26.3)	0.18	23.4(21.5–25.7)	24.2(22.4–26.4)	<0.001	23.7(21.9–25.9)	24.3(22.5–26.5)	<0.001
<25	289(56.0)	2130(60.5)	0.15	129(67.9)	2370(59.7)	0.07	607(64.6)	1892(58.7)	0.004
25–30	196(38.0)	1200(34.1)		51(26.8)	1385(34.9)		284(30.2)	1152(35.7)	
≥30	31(6.0)	190(5.4)		10(5.3)	217(5.5)		48(5.1)	179(5.6)	
Smoking status, n(%)			0.04			0.04			<0.001
Never-smoking	336(59.0)	2174(56.7)		138(61.3)	2452(56.7)		667(64.3)	1923(54.8)	
Former-smoking	84(14.7)	453(11.8)		33(14.7)	527(12.2)		148(14.3)	412(11.7)	
Current smoking	137(24.0)	1109(29.0)		46(20.4)	1243(28.8)		199(19.2)	1090(31.1)	
Unknown	13(2.3)	95(2.5)		8(3.6)	101(2.3)		24(2.3)	85(2.4)	
Heavy drink, n(%)	53(9.3)	463(12.1)	0.054	11(4.9)	517(12.0)	0.001	71(6.8)	457(13.0)	<0.001
Random glucose value on admission, median (IQR)	6.2(5.6–7.1)	6.1(5.2–6.7)	<0.001	6.2(5.6–7.2)	6.1(5.3–6.7)	0.01	6.2(5.5–7.2)	6.1(5.2–6.7)	<0.001
Pre-stroke mRS >1, n(%)	43(7.5)	130(3.4)	<0.001	23(10.2)	157(3.6)	<0.001	89(8.8)	91(2.6)	<0.001
NIHSS on admission, median(IQR)	2(1–3)	2(1–3)	0.047	2(1–3)	2(1–3)	0.64	2(1–3)	2(1–3)	<0.001
mean(SD)	1.7±1.1	1.8±1.0		1.8±1.1	1.7±1.0		1.9±1.0	1.7±1.0	
Subtype of stroke*, n(%)			0.001			<0.001			<0.001
Large-artery atherosclerosis	261(45.8)	1604(41.9)		103(45.8)	1812(41.9)		469(45.2)	1446(41.2)	
Small-artery occlusion	110(19.3)	926(24.2)		31(13.8)	1040(24.1)		202(19.5)	869(24.8)	
Cardioembolism	26(4.6)	85(2.2)		13(5.8)	102(2.4)		38(3.7)	77(2.2)	
Other or undetermined	18(3.2)	139(3.6)		7(3.1)	156(3.6)		29(2.8)	134(3.8)	
Unknown	155(27.2)	1077(28.1)		71(31.6)	1213(28.1)		300(28.9)	984(28.0)	
History of disease, n(%)									
Stroke	248(43.5)	1077(28.1)	<0.001	91(40.4)	1274(29.5)	<0.001	410(39.5)	955(27.2)	<0.001
Hypertension	400(70.2)	2419(63.1)	0.001	156(69.3)	2749(63.6)	0.08	710(68.4)	2195(62.5)	<0.001
Dyslipidemia	74(13.0)	422(11.0)	0.17	23(10.2)	487(11.3)	0.63	100(9.6)	410(11.7)	0.07
Coronary heart disease	114(20.0)	431(11.3)	<0.001	47(20.9)	519(12.0)	<0.001	166(16.0)	400(11.4)	<0.001
Atrial fibrillation	61(10.7)	170(4.4)	<0.001	26(11.6)	213(4.9)	<0.001	77(7.4)	162(4.6)	<0.001
Medications during hospitalization, n(%)									
Statins	265(46.5)	1760(45.9)	0.81	93(41.3)	1996(46.2)	0.16	453(43.6)	1636(46.6)	0.09
Antiplatelet	409(71.8)	2890(75.4)	0.06	151(67.1)	3261(75.4)	0.005	767(73.9)	2645(75.4)	0.34
Anticoagulation	14(2.5)	41(1.1)	0.006	5(2.2)	51(1.2)	0.28	12(1.2)	44(1.3)	0.80
Swallowing assessment, n(%)	222(39.0)	1352(35.3)	0.09	81(36.0)	1543(35.7)	0.93	395(38.1)	1229(35.0)	0.07
Pneumonia, n(%)	58(10.2)	144(3.8)	<0.001	49(21.8)	166(3.8)	<0.001	114(11.0)	101(2.9)	<0.001
Urinary tract infection, n(%)	13(2.3)	54(1.4)	0.11	7(3.1)	63(1.5)	0.09	27(2.6)	43(1.2)	0.002

IQR, interquartile range; mRS, modified Rankin Scale; NIHSS, National Institutes of Health stroke scale; and SD, standard deviation.

* Stroke subtype was defined by the Trial of Org 10172 in Acute Stroke Treatment (TOAST) classification.

**Table 3:** Comparison of the outcomes between the minor stroke patients with and without diabetes.

Time	Outcome	n/N (%)		Univariable analyses		Multivariable analyses	
		With DM	Without DM	OR	P value	Adj.OR (95% CI)	P value
3 m	Stroke recurrence	128/1202(10.7)	269/3243(8.3)	1.32	0.01	1.23(0.98–1.54) ^**†**^	0.08
	Death	32/1230(2.6)	85/3318(2.6)	1.02	0.94	0.95(0.62–1.45) ^‡^	0.79
	Poor outcome*	345/1230(28.0)	673/3318(20.3)	1.53	<0.001	1.46(1.24–1.71) ^§^	<0.001
1 y	Stroke recurrence	190/1192(15.9)	380/3209(11.8)	1.41	<0.001	1.31(1.08–1.59) ^**†**^	0.007
	Death	65/1230(5.3)	160/3318(4.8)	1.10	0.52	1.05(0.77–1.44) ^‡^	0.75
	Poor outcome*	358/1230(29.1)	680/3318(20.5)	1.59	<0.001	1.51(1.28–1.77) ^§^	<0.001

CI, confidence interval; DM, diabetes mellitus; and OR, odds ratio.

* Poor outcome: modified Rankin Scale 2–6

^†^ Adjusted for age, smoking status, history of stroke, pre-stroke modified Rankin Scale, National Institutes of Health stroke scale on admission, stroke subtype, history of hypertension, history of coronary heart disease, history of atrial fibrillation, anticoagulation use and pneumonia during hospitalization.

^‡^ Adjusted for age, body mass index, smoking status, heavy drink, history of stroke, pre-stroke modified Rankin Scale, stroke subtype, history of coronary heart disease, history of atrial fibrillation, antiplatelet therapy and pneumonia during hospitalization.

^§^Adjusted for age, gender, body mass index, smoking status, heavy drink, history of stroke, pre-stroke modified Rankin Scale, National Institutes of Health stroke scale on admission, stroke subtype, history of hypertension, history of coronary heart disease, history of atrial fibrillation, pneumonia and urinary tract infection during hospitalization.

### DM associated with poor prognosis of minor stroke subtypes

Because of the heterogeneity of minor stroke, we examined whether DM was associated with the prognosis of all subtypes or only specific subtypes. Among the 4,548 patients, 3,264 (71.8%) had data of TOAST classification, of whom 1,915 (58.7%) were LAA, 1,071 (32.8%) were SAO, 115 (3.5%) were CE, 54 (1.7%) were other determined pathogenesis, and 109 (3.3%) were undetermined pathogenesis. Baseline characteristics of patients according to stroke subtypes are presented in Table 4. Patients with LAA or SAO subtype had more risk factors of DM and hypertension, whereas those with CE subtype were older, more likely to be female, and had more risk factors of coronary heart disease and atrial fibrillation.

**Table 4:** Baseline characteristics of patients with different minor stroke subtypes.

Variable	LAA (N = 1915)	SAO (N = 1071)	CE (N = 115)	Others or undetermined (N = 163)	p value
Age (year), mean(SD)	64.4±11.7	64.1±11.7	68.8±11.9	58.3±15.2	<0.001
Female, n(%)	631(33.0)	374(34.9)	58(50.4)	59(36.2)	0.002
Body mass index (kg/m^2^), median (IQR)	24.4(22.5–26.4)	24.2(22.4–26.2)	23.8(22.2–26.2)	24.2(22.3–26.6)	0.37
<25	1012(57.9)	598(61.2)	60(63.8)	93(58.9)	0.23
25–30	636(36.4)	319(32.6)	33(35.1)	56(35.4)	
≥30	98(5.6)	61(6.2)	1(1.1)	9(5.7)	
Smoking status, n(%)					0.11
Never-smoking	1071(55.9)	621(58.0)	77(67.0)	91(55.8)	
Former-smoking	236(12.3)	141(13.2)	17(14.8)	18(11.0)	
Current smoking	554(28.9)	290(27.1)	20(17.4)	51(31.3)	
Unknown	54(2.8)	19(1.8)	1(0.9)	3(1.8)	
Heavy drink, n(%)	245(12.8)	120(11.2)	8(7.0)	21(12.9)	0.20
Random glucose value on admission, median (IQR)	6.1(5.3–6.9)	6.1(5.3–6.7)	6.1(5.5–7.0)	6.0(5.1–6.7)	0.03
Pre-stroke mRS >1, n(%)	81(4.2)	43(4.0)	3(2.6)	8(4.9)	0.59
NIHSS on admission, median(IQR)	2(1–3)	2(1–3)	2(1–3)	2(1–2)	0.36
mean(SD)	1.8±1.0	1.7±1.0	1.7±1.1	1.7±1.0	
Diabetes, n(%)	567(29.6)	304(28.4)	23(20.0)	38(23.3)	0.06
History of disease, n(%)					
Stroke	606(31.6)	302(28.2)	39(33.9)	41(25.2)	0.08
Hypertension	1293(67.5)	716(66.9)	68(59.1)	85(52.2)	<0.001
Dyslipidemia	210(11.0)	137(12.8)	14(12.2)	21(12.9)	0.48
Coronary heart disease	254(13.3)	119(11.1)	29(25.2)	23(14.1)	<0.001
Atrial fibrillation	69(3.6)	20(1.9)	91(79.1)	7(4.3)	<0.001
Medications during hospitalization, n(%)					
Hypoglycemic*	468(24.4)	251(23.4)	17(14.8)	34(20.9)	0.09
Antihypertensive	977(51.0)	562(52.5)	51(44.4)	65(39.9)	0.01
Statins	967(50.5)	524(48.9)	54(47.0)	67(41.1)	0.12
Antiplatelet	1515(79.1)	876(81.8)	72(62.6)	126(77.3)	<0.001
Anticoagulation	10(0.5)	7(0.7)	28(24.4)	3(1.8)	<0.001
Swallowing assessment, n(%)	717(37.4)	481(44.9)	48(41.7)	52(31.9)	<0.001
Pneumonia, n(%)	108(5.6)	41(3.8)	19(16.5)	5(3.1)	<0.001
Urinary tract infection, n(%)	37(1.9)	13(1.2)	4(3.5)	2(1.2)	0.21

CE, cardioembolism; IQR, interquartile range; LAA, large-artery atherosclerosis; mRS, modified Rankin Scale; NIHSS, National Institutes of Health stroke scale; SAO, small-artery occlusion; and SD, standard deviation.

* Hypoglycemic medications include use of insulin or oral hypoglycemic agents.

**Fig 2:**
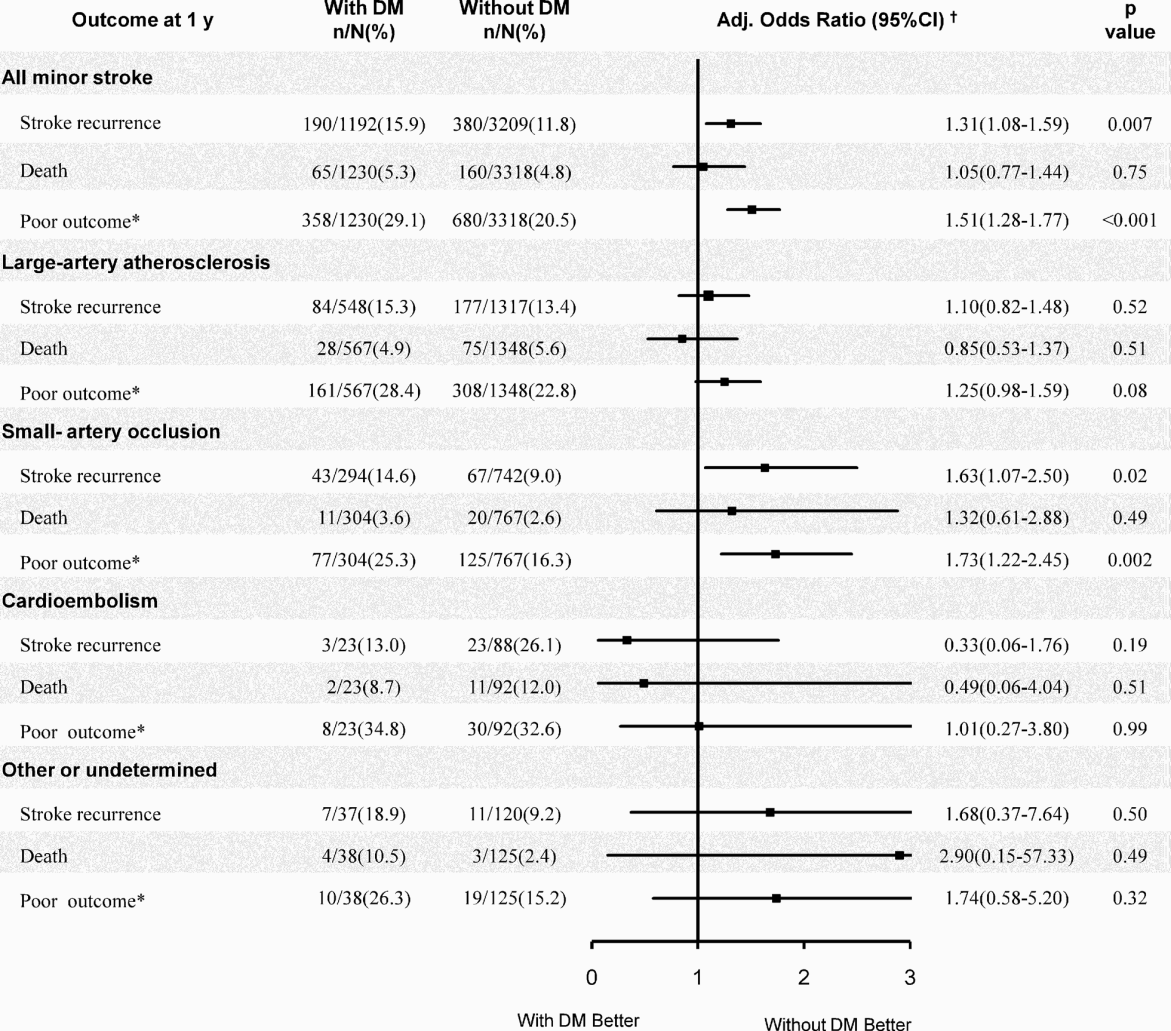
Adjusted OR of 1-year outcome for diabetes versus non-diabetes by stroke subtype. CI, confidence interval; DM, diabetes mellitus; and OR, odds ratio. * Poor outcome: modified Rankin Scale 2–6; † The same adjustment as corresponding multivariable analysis in Table 2 excluding stroke subtype for adjustment in subgroup analysis.

Fig 2 shows the associations of DM with the 1-year outcome of minor stroke according to stroke subtype. DM was only significantly associated with stroke recurrence (OR, 1.63; 95%CI: 1.07–2.50) and poor outcome (OR, 1.73; 95%CI: 1.22–2.45) in SAO subgroup, but not in the other subgroups (Fig 2).

### DM associated with poor prognosis of minor stroke and its subtypes after adjusting for random glucose on admission

As Fig 2 indicated, DM was associated with some unfavorable prognosis after the minor stork, we tried to dissect the linkage underlying the association by entering the random glucose on admission into the multiple analysis model (Table 5). With random glucose as a covariate incorporated into the model, both DM (OR, 1.38; 95%CI: 1.15–1.66) and random glucose (OR, 1.04; 95%CI: 1.00–1.07) were significantly associated with 1-year poor outcome after stroke onset. In SAO subtype, with random glucose as a covariate in the model, DM (OR, 1.66; 95%CI: 1.13–2.44) but not random glucose (OR, 1.02; 95%CI: 0.94–1.11) was significantly associated with 1-year poor outcome.

**Table 5:** Association of DM with prognosis of minor stroke or its subtypes after adjusting for random glucose on admission.

Outcome at 1 y	DM		Random glucose	
	Adj.OR^†^(95% CI)	p value	Adj.OR^†^(95% CI)	p value
**All minor stroke**				
Stroke recurrence	1.23(0.99–1.53)	0.07	1.03(0.98–1.07)	0.22
Poor outcome*	1.38(1.15–1.66)	<0.001	1.04(1.00–1.07)	0.05
**Small-artery occlusion subtype**				
Stroke recurrence	1.52(0.94–2.48)	0.09	1.03(0.93–1.14)	0.57
Poor outcome*	1.66(1.13–2.44)	0.01	1.02(0.94–1.11)	0.64

CI, confidence interval; DM, diabetes mellitus; and OR, odds ratio.

* Poor outcome: modified Rankin Scale 2–6

† DM and random glucose on admission were included in the same multivariable logistic regression model, in which other adjusted covariates were same as that in Fig 2.

## Discussion

In this large-scale national stroke registry study, we found that DM increased the risk of stroke recurrence and poor outcome of minor stroke at 1 year’s follow-up, whereas these associations were only found in the SAO subtype, but not in other subtypes of minor stroke. Furthermore, our study also demonstrated that hyperglycemia status may not be the only factor of DM per se contributing to the unfavorable prognosis of minor stroke.

Few studies investigated the risk factor of recurrent stroke or prognosis after minor stroke [2,3,22]. Previous studies that focused on minor stroke showed a negative association between DM and recurrent ischemic events [2] or poor outcome [3]. Our study showed that the association between DM and recurrence of stroke after minor stroke was similar with the pattern in patients with general stroke [7] or lacunar stroke [23]. The difference between our study and the previous studies may be explained by the small sample size and different definition of outcomes in the previous studies (Zhang C et al [2] used recurrent ischemic stroke or TIA; Sato S et al [3] used poor outcome defined as an mRS score of 3 to 6 at 90 days). Data from the Austrian Stroke Unit Registry also showed that DM was an independent risk factor for early clinical deterioration in patients with TIA or minor stroke [22].

Our study indicated that like hypertension [20], the positive association between DM and stroke recurrence was only found in the SAO subtype of minor stroke. DM or hyperglycemic status may underlie increased arterial stiffness and impaired endothelial function of stroke patients, which may contribute to occurrence of stroke of the SAO subtype [24,25]. A population-based cross-sectional study [13], cohort study [14] and a meta-analysis [15] showed that DM was an independent risk factor of occurrence of stroke with SAO subtype, but this was not validated in another cross-sectional study [26] and cohort study [27]. Our study did not show a significant association between DM and stroke recurrence in the LAA subtype. This was supported by some [14,27], but not all [13,15,26], previous studies that also did not showed DM to be an independent risk factor of occurrence of stroke with LAA subtype. Besides, DM was also found to contribute to the poor outcome of minor stroke patients with SAO subtype in our study. A previous study showed that SAO patients with history of diabetes had higher risk of unfavorable outcome at 3 month after intravenous tissue-type plasminogen activator (tPA) treatment [28]. Our large-scale prospective observational study may add to the evidence that diabetes is a risk factor for stroke recurrence and poor outcome in the SAO subtype of minor stroke.

Our study showed that 27% of minor stroke patients had DM, which was higher than that in the general population (11.6% in Chinese adults) [29]. Given the high prevalence of DM in patients with minor stroke and high risk of stroke recurrence and unfavorable outcome after minor stroke [3,4], our results may have implications for appropriate antidiabetic treatment for minor stroke patients, especially for those with SAO subtype. In our study, we also found that DM status was still associated with unfavorable prognosis of minor stroke or its subtype after serum glucose and other potential risk factors for the outcome of the minor stroke had been adjusted. This result indicates that hyperglycemia may not be the only factor of DM per se that contributed to the unfavorable prognosis of minor stroke [7]. A previous study showed that in addition to the hyperglycemia status of DM per se, hyperinsulinemia also cause oxidative stress on the endothelial and endothelial progenitor cells, which is an important underlying pathogenesis for stroke [6]. Our study suggests that elimination of the hyperglycemia alone could not efficiently enhance the prognosis of minor stroke patients with DM; a concurrent and comprehensive strategy to improve insulin resistance should be encouraged.

Our study has some limitations. First, all the participating hospitals in the CNSR study were from the urban areas of China which might be hospital selection bias. It is not known whether the prevalence of DM in patients with minor stroke and the distribution of stroke subtypes in hospitals from the rural areas were similar to those that participated in our study. Second, the definition of DM in this analysis was based on self-reported history of physician diagnosis of DM at admission or hypoglycemic agents use during hospitalization and we did not collect new cases of DM after discharge which may have impact on the results. Third, because serum insulin was not available in the CNSR, we could not identify the precise action of hyperglycemia status alone on the prognosis of minor stroke. Finally, changes in medical services over time might have influenced the study results.

## Conclusions

In this large cohort observational study, we found DM significantly increased the risk of stroke recurrence and poor outcome in the SAO subtype of minor stroke, but not in other subtypes of minor stroke.
